# Medicine Dose Adjustment Practice and Associated Factors among Renally Impaired Patients in Amhara Regional State, Ethiopia

**DOI:** 10.1155/2021/8238250

**Published:** 2021-12-01

**Authors:** Tirsit Kestela Zeleke, Tilahun Yemanu Birhan, Ousman Abubeker Abdela

**Affiliations:** ^1^Department of Pharmacy, College of Medicine and Health Sciences, Debre Markos University, P.O. Box 269, Debre Markos, Ethiopia; ^2^Department of Epidemiology and Biostatistics, Institute of Public Health, College of Medicine & Health Sciences, University of Gondar, P.O. Box 196, Gondar, Ethiopia; ^3^Department of Clinical Pharmacy, School of Pharmacy, College of Medicine and Health Science College, University of Gondar, P.O. Box, Ethiopia

## Abstract

**Background:**

Kidney disease affects absorption, distribution, metabolism, and excretion of medicines and their metabolites. Therefore, when prescribing medicines for patients with kidney disease, dose adjustment is an accepted standard of practice.

**Objective:**

This study aimed to assess medicine dose adjustment practice and associated factors among adult patients with renal impairment admitted to medical wards at Amhara region referral hospitals.

**Method:**

Multicenter, institution-based, cross-sectional study was conducted from March 28, 2020, to August 30, 2020. The data was collected by using a pretested interviewer-administered structured questionnaire. Data were entered into Epi-Data version 4.6 and transferred into SPSS version 25 for further data processing and analysis. Descriptive statistics such as frequencies and percentages were computed. Both bivariable and multivariable binary logistic regression analyses were fitted to identify factors associated with dose adjustment practice. A 95% confidence interval and a *p* value less than 0.05 were used to declare statistical significance.

**Result:**

Among 815 medicines' prescriptions that needed dose adjustment, 417 (51.2%) of them were dosed inappropriately. Number of medicines, number of comorbidities, and being unemployed were significantly associated with inappropriate dose adjustment.

**Conclusion:**

Our study revealed that there was a considerable rate of inappropriate dose adjustment in patients with renal impairment. Training for health care providers, use of guidelines, and communication with clinical pharmacists should be encouraged for good prescription practice.

## 1. Introduction

Kidney is the main organ behind maintaining homeostasis, acid-base balance, and optimal electrolytes balance [1]. It has also an important role in the disposition of medicines and other toxic substances [2]. It can be malfunctioned by diseases including, autoimmune disorders, diabetes mellitus, and infection of various origin [3].

Renal impairment is an anomalous kidney function where the kidney cannot discharge or wipe out toxic substances enough from the body [1]. Acute kidney injury (AKI) will happen when there is an abrupt (within 48 hours) reduction in kidney function, which is defined as an absolute increase in serum creatinine of ≥0.3 mg/dl (≥26.4 *μ*mol/l) or a 50% increase in serum creatinine (1.5-fold) from baseline [4]. Chronic kidney disease (CKD) is defined as glomerular filtration rate (GFR) of less than or equal to 60 mL/min per 1·73 m^2^, or markers of kidney damage, or both, of at least three-month duration, irrespective of the underlying cause [5]. Both AKI and CKD can cause trouble on multiple organ systems and these physiological changes have been correlated with significant alterations in the pharmacokinetics and the pharmacodynamics of many medicines [6].

Kidney disease affects absorption, distribution, metabolism, and excretion of medicines and their metabolites [7].The main problems with medicines or their metabolites in renal impairment is failure to be excreted [8]. Many side effects of medicines are poorly tolerated by patients with renal impairment and some medicines ceasing to be effective when renal function is reduced [9]. Therefore, when prescribing medicines for patients with kidney disease, multiple factors must be taken into account [10]. Among these, dose adjustment of medicines is an accepted standard of practice [11]. If doses are not adjusted appropriately, accumulation and toxicity can develop promptly [12].Therefore, dose adjustment should be considered when medicines are prescribed to patients with renal impairment [13].

Creatinine clearance (CrCl) is used to measure glomerular filtration rate (GFR), a measurement of renal clearance of a particular substance from the plasma, in routine clinical practice and is reported as plasma volume cleared of creatinine (ml) per time (min) [14].

After assessing renal function, dose adjustment can be accomplished either by decreasing dose or by prolongation of administration interval [15]. For medicines whose toxicity correlate with a rapid rise in plasma concentration the dose interval should be prolonged, which provides a constant plasma medicine concentration without increasing the risk of nephrotoxicity [16].

According to the 2011 WHO data, kidney disease in Ethiopia accounted for 1.47% of total deaths and is predicted to raise [17]. A cross-sectional study from Tikur Anbessa Specilaised Teaching Hospital, Addis Ababa, Ethiopia, reported that more than half of the doses of medicines prescribed for patients with renal impairment admitted to medical wards were inappropriate [12]. This study aimed to explore the practice of medicine dose adjustment for patients with renal impairment in North-West part of Ethiopia.

## 2. Material and Methods

### 2.1. Study Design

An institution-based, cross-sectional, observational study was conducted.

### 2.2. Study Area and Period

The study was conducted at selected referral hospitals in Amhara regional state from March 28, 2020, to August 30, 2020. The state is the second largest region in Ethiopia with six referral hospitals. Among these, three of them (Tibebe Gion, Felege Hiwot, and Debre Markos referral hospitals) were selected randomly.

Felege Hiwot comprehensive referral hospital is a referral hospital located in Bahir Dar, 562 km North-West of Addis Ababa. It delivers health care services with medical, surgical, gynecological, orthopedic, intensive care units, pediatrics, and ophthalmological wards with a total of 400 beds capacity and around 15 adult outpatient departments and 561 staffs. It serves a catchment population of more than 7 million, and about 500 clients visit the hospital daily [18–20]. Internal medicine ward contains 85 beds; on average, 41 patients with renal impairment were admitted to the medical ward monthly. There is also a dialysis center that contains 12 beds.

Debre Markos Referral Hospital is a referral hospital found in Debre Markos town, and it is the only referral hospital found in East Gojjam Zone, which is 300 km from Addis Ababa. It currently serves a population of around 3.5 million in its catchment area among medical wards [21, 22]. Internal medicine ward had 40 beds; 1648 patents were admitted to medical wards annually. Tibebe Ghion specialized hospital is relatively new hospital located at the southern end of the Bahir Dar city. The hospital delivers health care services with medical, surgical, gynecological, intensive care units, and pediatrics [23].

### 2.3. Population

#### 2.3.1. Source Population

All patients with renal impairment were admitted to medical wards at the selected hospitals.

#### 2.3.2. Study Population

All patients with renal impairment were admitted to medical wards at the selected hospitals during data collection period.

### 2.4. Inclusion and Exclusion Criteria

#### 2.4.1. Inclusion Criteria

Patients admitted to medical wards with diagnosis of either AKI or CKD during the study period, age 18 years and above, receiving at least one pharmacological agent, and who had at least one estimated GFR value of ≤60 mL/min/1.73 m^2^ were included in this study.

#### 2.4.2. Exclusion Criteria

Patients who were critically ill, with severe psychiatric illness, and with incomplete medicine record were excluded from the study.

### 2.5. Sample Size and Sampling Technique

#### 2.5.1. Sample Size Determination

The sample size was estimated by using single-population proportion formula: considering the assumptions of 95% confidence interval, prevalence of inappropriate dose adjustment was 51% (0.51) [12]. The desired margin of error at 5% was calculated as follows:

 
*n*=*z*2*p*(1 − *p*)*d*2
 
*n*=(1.96)2(0.51)(0.49)=384(0.05)2

Finally, we had 10% nonresponse rate, which made our final sample size to be 424.

#### 2.5.2. Sampling Technique

All the first 424 patients who were admitted to medical wards of the selected hospitals during the study period and fulfill the inclusion criteria were included.

### 2.6. Study Variables

#### 2.6.1. Dependent Variable

The dependent variable was dose adjustment appropriateness.

#### 2.6.2. Independent Variables

Age, sex, weight, residence, education status, occupation, presence of comorbidities and complications, number of comorbidities and complications, stage and severity of the disease, number of prescribed medicines, prescriber's specialty, and length of service were independent variables.

### 2.7. Operational Definitions

Renal impairment: it is a medical condition in which the kidneys fail to adequately filter waste products with estimated GFR ≤60 mL/min/1.73 m^2^) [24].

Appropriate dose adjustment: when the medicine dose is adjusted based on the patient's CrCl as recommended by the guidelines “Medicine Prescribing in Renal Failure: Dosing Guidelines for Adults and Children” [25].

Inappropriate dose adjustment: when the dose prescribed is not in conformity to the patient's CrCl as recommended by “Medicine Prescribing in Renal Failure: Dosing Guidelines for Adults and Children [25].

### 2.8. Data Collection Procedure and Quality Control

#### 2.8.1. Data Collection Tools and Procedures

Data were collected by a pretested interviewer-administered structured questionnaire. The questionnaire was prepared by the investigators after reviewing different literatures [10, 12, 26–28]. Five trained BSc: nurses participated in the data collection process where three of them were data collectors and two of them were supervisors. Patients' sociodemographic characteristics were collected by asking them, the weight of patients was found by measurement, and clinical parameters such as stage of CKD, SrCr level, and medicines prescribed were taken from patient's medical chart. Length of service and educational status of prescribers were obtained by asking them.

Recent SrCr value recorded prior to medicine prescription ≥1.2 mg/dl used as a cutoff point in preselection of patients. Then, for those whose SrCr level ≥1.2 mg/dl, GFR was calculated by using CG formula patients; then, GFR value of ≤60 mL/min/1.73 m^2^ was included in the study.

The estimated GFR (eGFR) was calculated using CG equation as follows [29]:

  Male: eGFR ml/min = [(140 − age (in year)) × weight (kg)]
  SrCr (mg/dl) × 72
  Female: eGFR ml/min = [(140 − age (in year) × weight (kg)] × 0.85
  SrCr (mg/dl) × 72

At the end, the doses of medicines assessed for appropriateness individually for each and every patient using dose adjustment guideline “Medicine Prescribing in Renal Failure: Dosing Guidelines for Adults and Children [25].

#### 2.8.2. Data Quality Assurance

Initially the data collection tool was developed in English after reviewing literatures. The tool had five main parts. The first part is intended to get information on sociodemographic characteristics of willing participants. The second section was meant to extract participants' clinical and laboratory values from medical records. The third section is intended to clarify prescribers' level of specialty and length of service. The fourth section is meant to extract medicines regimen prescribed. The last section was designed to put assessment results of appropriateness of medicines dose adjustment practice of medicines that guide lines recommend dose adjustment. The first section of the tool was translated to Amharic language as it and then back to English language to keep its consistency. The Amharic version was used to directly ask patients and fill the questionnaire. However, information to be filled on sections two to five were available on patients' medical record with English language. Therefore, there was no need to translate them to local language.

A pretest was done on 21 patients (5% of the calculated sample size) with similar diagnosis who were admitted and treated at university of Gondar comprehensive and specialized hospital for the assessment of the questionnaire clarity and sociocultural compatibility. Data from participants of the pretest were not included in the final analysis. The hospital was found at Amhara regional state, in which there were similar demographics, clinical characteristics of the population. The pretest helped to modify the original data extraction tool formulated to suit to the objective of the study.

Training was given to both the data collectors and supervisors by the principal investigator about the purpose of the study, data collection procedures and ethical issues to follow during data collection process.

Furthermore, the supervisors made on-site supervision during the whole period of data collection and the collected data were reviewed and checked for completeness and consistency by the investigator.

### 2.9. Data Management and Analysis

The completeness and consistency of the data was checked, coded and entered into the Epi-data4.6 then exported to SPSS 25 for further analysis. Descriptive statistics done using frequencies and percentages for categorical variables while summary measures for continues variable. Hosmer and Lemeshow test for goodness of fitness used for the model fit of each variable to use in logistic regression model. Bivariable and multivariable logistic regression was fitted in order to see the effect of independent variables on the dose adjustment practice.

Variables having *p* value less than 0.2 in the bivariable analysis were transferred to multiple logistic regression models to adjust for confounder's effects, and those variables with *p* value <0.05 were considered as significantly associated in the final model.

Adjusted odds ratio (AOR) with 95% confidence interval (CI) was computed and interpreted accordingly.

Furthermore, multicollinearity between the explanatory variables was assessed with variance inflation factor (VIF) to identify and avoid redundant variables that may affect our estimate; the VIF was in the acceptable range (1–5) [30].

Finally, the result of the study was presented using tables, figures, and texts based on the outcome.

### 2.10. Ethical Considerations

Ethical clearance was obtained from university of Gondar college of medicine and health Sciences on March 23/2020 (Ref No. SOP/065/2020). Permission letter was obtained from Felege Hiwot, Debre Markos, and Tibebe Gion referral hospitals. The nature of the study was fully explained to the study participants and informed verbal consent was obtained. Any personal identifiers were not included in the questionnaire to keep the confidentiality of the information. On top of this, COVID-19 protective measures were used by data collectors and supervisors.

## 3. Result

### 3.1. Sociodemographic and Clinical Characteristics of Study Participants

There were a total of 2850 patients admitted to medical wards, during the study period from March 2020 to August 2020. Among these, 950 patients had SrCr value of ≥1.2. Based on inclusion criteria, a total of 424 (19.7%) patients with GFR ≤60 were considered for evaluation. In this study, 424 patients were participated with 100% response rate. The mean age of the participants was 47.5 years with a SD of ±16.6. More than half of the patients were male 227 (53.5%). Mean weight for the study participants (*n* = 424) was 60.35 kg with a SD of ±9.3.223 (52.6%) patients were from urban. Majority of participants 189 (44.6%) had no formal education. More than half of participants were employed 264 (62.3%) (Table 1).

From the total respondents, 374 (88.2%) of them had comorbidity and complications. Specifically, 158 (37.3%) of them were hypertensive, 124 (29.2%) were diabetic, and 99 (23.3%) of participants had congestive heart failure. Furthermore, it was observed that among CKD patients, 53 (12.5%) had CKD stage three (Table 2).

Among 1581 medicine prescriptions, 1334 (84.4%) were prescribed by general physicians. Furthermore, majority of medicine prescriptions (1122 (71%)) were prescribed by physicians who had work experience of ≥3 years (Table 3).

### 3.2. Frequency of Medicines Prescription in Patients with Renal Impairment

There were total of 1581 medicine prescriptions for the 424 enrolled patients with a mean of 3.7 medicine prescription per patient. A range of 1 to 9 medicines were prescribed per patient. Furosemide was the most frequently prescribed medicine type which was ordered 307 times, followed by enalapril, cimetidine, vancomycin, and nifedipine, which were prescribed 167, 89, 87, and 81 times, respectively.

Among medicines being recommended for dose adjustment, enalapril was given frequently and ranked the highest followed by cimetidine, vancomycin, and spironolactone (Table 4).

### 3.3. Appropriateness of Dose Adjustment in Patients with Renal Impairment

From the 1581 medicine prescriptions made, 815 (51.5%) of them are medicines that need to undergo dose adjustment. From the medicine prescriptions that needed dose adjustment, 398 (48.8%) of them were adjusted appropriately.

Among medicines that need dose adjustment, cimetidine was the most commonly inappropriately adjusted medicine 63 (70.7%) followed by atenolol, ciprofloxacin, and ceftazidime, while metformin was the least with inappropriate dose adjustment (Table 5).

### 3.4. Factors Associated with Dose Adjustment Practice in Patients with Renal Impairment

Bivariable and multivariable analysis showed that age, sex, weight, education, residence, SrCr, CrCl, presence of comorbidity, stage of renal impairment, length of service, and specialty of prescribers did not have significant effect on dose adjustment practice.

However, dose adjustment practice was significantly associated with number of medicines (AOR = 3.20 (2.28–4.49)); that is, as the number of prescribed medicines increases, the odds of inappropriate dose adjustment increased by 3.20 times. Regarding occupational status, being unemployed increases the odds of inappropriate dose adjustment practice by 3.18, AOR = 3.18 (95% CI 1.45–7.01) as compared to employed one. Also, as the number of comorbidities increases the odds of inappropriate dose adjustment increases by 1.65, AOR = 1.65 (95% CI 1.09–2.48) (Table 6).

## 4. Discussion

This study found that the prevalence of renal impairment among patients admitted to medical wards at Amhara region referral hospitals, Ethiopia, was 19.7%, which was appreciably higher than previously reported study in the same country in 2015 which was found to be 9% [12]. Although an eGFR of <59 mL/min/1.73 m^2^ was used in their study to define renal impairment compared to eGFR ≤60 mL/min/1.73 m^2^ used in our study, the prevalence of renal impairment was less. The definition of renal impairment varies among different information sources [10]. However, medicine dosing guidelines are typically derived from studies performed in patients with stable, chronic renal insufficiency and the recommendations are extrapolated to seriously ill patients with acutely decreased renal function [25]. For the purpose of this study to evaluate the appropriateness of medicine dosing adjustment, renal impairment was defined as estimated GFR ≤60 ml/min/1.73 m^2^ [24]. The possible explanation for this discrepancy may be due to the current increasing number of patients visiting hospitals and the prevalence of renal impairment increases in an alarming rate [31], which is less prevalent than recent study done in Botswana that showed 29% [10]. This may be attributed to the fact that we used estimated GFR, rather than a SrCrto-defined renal impairment. As a result of this, it is likely that we may have missed some patients with renal impairment.

The mean estimated GFR of the population group studied in our study was 32.71 ml/min/1.73, which is higher than a study from Mekelle, Ethiopia, with a mean eGFR of 28.84 mL/min/1.73 m which used MDRD equation to calculate GFR [32]. This difference could possibly be due to the different equations used [33].

The mean of medicines prescribed to patients with renal impairment was 4. The higher number of medicines prescribed in our study may partly be explained by the fact that 88.2% of patients with medicines that required dose adjustments had comorbidities and complications with an expected increased pill burden. Polypharmacy in patients with renal impairment has been found across many similar studies [12, 27].

The total number of prescriptions that required dose adjustment and the percentage of inappropriate dosing varied in different studies [1, 2, 10, 12, 26, 27, 32]. The current study showed that 51.2% of the medicines that required adjustment were not adjusted appropriately; only 48.8% had correctly recommended doses, in line with a study conducted in Addis Ababa, Ethiopia, which reported that from 372 prescriptions, 51% prescription entries requiring dose adjustment were found to be in appropriate [12], and in Mekelle, Ethiopia, among 360 orders that need adjustment 51% were inappropriate [32]. Also comparable results found with studies done in Saudi Arabia, South Africa, and Botswana, which showed inappropriate dose adjustment in 53.1%,53.0%, and 54.3% of prescriptions, respectively [2, 10, 27]. This similarity may be due to the reason that these health care facilities are similar in structure, since they are referral and academic hospitals found in developing countries.

Compared to studies conducted in Lebanon, Pakistan, and Palestine, lower inappropriate dose adjustment practice was found, which showed 63%, 58.2%, and 73.58% of prescriptions were inappropriately adjusted, respectively [26, 28, 34]; this result is quite encouraging.

Higher inappropriate dose adjustment compared to study conducted in France, Australia, and Indonesia, which showed that 34%, 44.8%, and 20.0% of the prescribed medicines dose were inappropriately adjusted, respectively [35–37].The better dose adjustment practice in these developed countries could be due to the incorporation of advanced computerized dose adjustment systems of reporting renal function, which help prescribers to know the need for dose adjustment [38] and also involvement of clinical pharmacists in their clinical settings that perhaps resulted in lower inappropriate dosing [39].

The practice of inappropriate dose adjustment found in our study is considered to be high. Several reasons may be elucidated to inappropriate medicine dosing in renal impairment [40, 41]. These include physicians' underestimation of renal function; commonly, they use serum creatinine level alone to assess renal function, which is often inappropriate to estimate actual renal function and leads to underestimation of renal impairment. Estimation of renal function is important when prescribing medicines that are known to be excreted renally to avoid inappropriate dose [25, 42]. If SrCr is used alone for medicine dose adjustment without CrCl, patients are exposed to an increased risk of adverse medicine reactions because renal function can be considerably impaired despite normal serum creatinine [43]. There was lack of reviewing renal function tests before prescribing, which was indicated by the high number of exclusions due to missing SrCr level in our patients' medical charts. On the top of this, physicians' limited knowledge about medicines that required dose adjustment which was revealed here by the high number of medicines not adjusted appropriately and some not adjusted at all. The other reason is although guidelines and recommendations that include lists of contraindicated medicines and those requiring dose adjustment are available [44–46]; rates of noncompliance with dosing guidelines and prescription of contraindicated medicines are common in patients with renal impairment [47]. Moreover, this finding is supported by our qualitative result. Although all clinical practitioners declared they adjust dose for patients with renal impairment, they stated that there is inappropriate dose adjustment due to various reasons; they mentioned that due to the lack of availability of updated books and guidelines, they simply adjust dose, depending on various and different methods, which may result in inconsistency and inappropriate dosing.

In addition, this study found that most frequently prescribed medicines with inappropriate dosing were cimetidine, atenolol, ciprofloxacin, ceftazidime, hydrochlorothiazide, vancomycin, and enalapril. These results are in line with previous studies [12] with the exception of enalapril that was prescribed more appropriately in their setting. Digoxin, metformin, and spironolactone were also inappropriately adjusted, which is comparative with a study done in Indonesia [36]. These findings show that there is underestimation of the adverse outcomes associated with several important medicines, such as cimetidine, atenolol, and antibiotics that are reported to induce nephrotoxicity.

When assessing patients' factors association with inappropriate dose, adjustment of the age and sex of patients were not associated. These results were in line with studies done in South Africa and Pakistan [27, 28]. Although the stage of renal impairment had no significant association, in our study, previous studies suggested stage of renal impairment and the presence of comorbidities were predictors of inappropriate dose adjustment [28]. This may be due to the fact that when there is comorbidity and complication the need for use of medicines will increase to treat them; in addition to this, when severity or stage in renal impairment progressed, complications become prevalent.

The number of medicines prescribed per patient was confirmed to be a statistically significant risk factor that increased the likelihood of inappropriate dose adjustment, which was comparable with study done in Korea, Botswana, and South Africa [1, 10, 27]. This may be owning to a reason as the number of medicines prescribed increased it may be difficult to check and monitor dose adjustment for each and every medicine. The other factor which was significantly associated was the number of comorbidities, patients with high number of comorbidities were more susceptible for inappropriate dose adjustment, which was in line with study conducted in South Africa [27]. This is due to the fact that when the number of comorbidities increases, the need for medicines increase which in turn increases the number of medicines that leads to inappropriate dose adjustment. Moreover, this finding is also supported by our qualitative result, in which participants mentioned that in patients with many medicines, it may be difficult to follow dose of all medicines that need adjustment and to address inappropriate dose adjustments since there is a high workload.

Another associated factor was occupation; patients who were unemployed were more prone to inappropriate dose adjustment.

### 4.1. Limitations

Despite interesting findings elicited in this study, we are aware of some limitations with this study. First, in the absence of a concise definition of renal impairment in the literature, we used an estimated GFR of ≤60 mL/min/1.73 m^2^, which corresponds to CKD stage 3. Some studies have used this exact definition; however, we are aware of other studies that have used absolute serum creatinine values or lower cutoffs of estimated GFR. Second, most dosing guidelines use stable GFR; some of the patients in this study had AKI making their serum creatinine/GFR unstable. GFR may become extremely difficult to estimate and unreliable in critically ill patients who experience rapidly changing renal function [48]. Finally, prescribers may have referred to guidelines that are different from the ones we used in our study.

## 5. Conclusion

This study concluded that inappropriate dose adjustment is common in patients with renal impairment admitted to medical wards. More than half of medicines prescribed requiring dose adjustment were inappropriately adjusted. Factors found to be significantly associated with dose adjustment practice were a higher number of prescribed medicines, higher number of comorbidities, and occupation of patients that is being unemployed. Therefore, attention should be paid to the abovementioned significant predictors of medicine dosing errors, and thus the doses of medicines should be prescribed carefully and appropriately to avoid the risk of medicine related toxicities and adverse outcomes.

## Figures and Tables

**Table 1:** Sociodemographic characteristics of patients with renal impairment admitted to medical wards at Amhara region referral hospitals, Amhara Ethiopia, in 2020 (*n* = 424).

Variables	Frequency	Percent
Age		
<20	13	3.1
20–40	142	33.5
41–60	175	41.3
>60	94	22.1

Sex		
Male	227	53.5
Female	197	46.5

Residence		
Rural	201	47.4
Urban	223	52.6

Education		
No formal education	189	44.6
Primary	72	17.0
Secondary	49	11.6
Higher	114	26.8

Occupation		
Employed	264	62.3
Unemployed	160	37.7

**Table 2:** Clinical characteristics of patients with renal impairment admitted to medical wards at Amhara region referral hospitals, Amhara, Ethiopia, in 2020 (*n* = 424).

Variables	Frequency	Percent
Reason for admission		
AKI	283	66.7
CKD	141	33.3

Presence of comorbidities		
Yes	374	88.2
No	50	11.8

Number of comorbidities		
CHF	99	23.3
HTN	158	37.3
Stroke	40	9.4
IHD	43	10.1
DM	124	29.2
Pneumonia	70	16.5
UTI	58	13.7
AGN	38	9.0
SSI	20	4.7
Sepsis	24	5.7
Asthma	58	13.7
Anemia	50	11.8
Others ^*∗*^	15	3.5

Stage in CKD patients		
Stage 3	53	12.5
Stage 4	37	8.7
Stage 5	52	12.3
	Mean	SD
Weight	60.35	9.3
Serum creatinine	3.03	2.4
GFR	32.71	14.7

^*∗*^Preeclampsia, deep vein thrombosis (DVT), epilepsy, appendicitis, and meningitis.

**Table 3:** Physicians' characteristics for medications prescribed in patients with renal impairment admitted to medical wards at Amhara region referral hospitals, Amhara Ethiopia, in 2020 (*n* = 424).

Variables	Frequency	Percent
Specialty		
General physician	1334	84.4
Internist	247	15.6

Length of work experience		
≤1 year	190	12
>2 years	269	17
≥3 years	1122	71

**Table 4:** Frequency of medications prescription in patients with renal impairment admitted to medical wards at Amhara region referral hospitals, Amhara, Ethiopia, in 2020 (*n* = 424).

Type of medications prescribed	Frequency of prescription
Enalapril	167
Cimetidine	89
Vancomycin	87
Spironolactone	80
Ciprofloxacin	74
Insulin	65
Hydrochlorothiazide	58
Ceftazidime	55
Digoxin	53
Metformin	49
Atenolol	38
Aspirin	51
Furosemide	307
Azithromycin	13
Ceftriaxone	69
Metronidazole	25
Amlodipine	25
Nifedipine	59
Metoprolol	49
Omeprazole	33
Atorvastatin	46
Warfarin	22
Others ^*∗*^	67

Others ^*∗*^ = heparin, ketoconazole, haloperidol, carvedilol, simvastatin, valproic acid, iron sulphate, propylthiouracil, amoxicillin, cobalamin, folic acid, beclomethasone puff, amitriptyline, hydralazine, diphenhydramine, paracetamol, carvedilol, and bisacodyl.

**Table 5:** Appropriateness of medicines dosage adjustment in patients with renal impairment, who were admitted to medical wards at Amhara region referral hospitals, Ethiopia, in 2020 (*n* = 424).

Type of medication	Frequency of prescription	Appropriately adjusted		Inappropriately adjusted	
		Frequency	Percent	Frequency	Percent
Cimetidine	89	26	29.3	63	70.7
Atenolol	38	13	34.3	25	65.7
Ciprofloxacin	74	26	35.2	48	64.8
Ceftazidime	55	21	38.2	34	61.8
Hydrochlorothiazide	58	25	43.2	33	56.8
Vancomycin	87	42	48.3	45	51.7
Digoxin	53	27	51.0	26	49.0
Spironolactone	80	42	52.5	38	47.5
Insulin	65	36	55.4	29	44.6
Enalapril	167	105	62.9	62	37.1
Metformin	49	35	71.5	14	28.5

**Table 6:** Factors associated with dose adjustment practice in patients with renal impairment admitted to medical wards at Amhara region referral hospitals, Amhara, Ethiopia, in 2020 (*n* = 424).

Variable	Inappropriate dose adjustment		Univariable analysis			Multivariable analysis		
	Yes	No	*p* value	COR	95% CI	*p* value	AOR	95% CI
*Age*								
<20	5 (38.5)	8 (61.5)		1			1	
20–40	60 (42.3)	82 (57.7)	0.79	1.17	(0.36–3.75)	0.17	5.0	(0.49–52.1)
41–60	88 (50.3)	87 (49.7)	0.41	1.61	(0.50–5.14)	0.41	2.6	(0.25–27.4)
>60	57(60.6)	37 (39.4)	0.13	2.46	(0.74–8.11)	0.88	0.8	(0.76–9.2)

*Sex*								
Male	103 (45.4)	124 (54.6)		1			1	
Female	107 (54.3)	90 (45.7)	0.06	1.43	(0.97–2.1)	0.88	0.95	(0.47–1.90)

*Residence*								
Rural	117 (58.2)	84 (41.8)	0.01	1.94	(1.32–2.86)	0.81	0.91	(0.41–2.01)
Urban	93 (41.7)	130 (58.3)		1			1	

*Occupation*								
Employed	109 (41.2)	155 (58.8)		1			1	
Unemployed	101 (63.1)	59 (36.9)	<0.001	2.43	(1.62–3.64)	0.04	3.18	(1.45–7.01) ^*∗*^

*Education*								
No formal education	119 (62.9)	70 (37.1)	<0.001	3.68	(2.25–6.03)	0.91	2.39	(0.86–6.61)
Primary	39 (54.1)	33 (45.9)	0.02	2.56	(1.39–4.70)	0.61	1.30	(0.46–3.61)
Secondary	16 (32.6)	33 (67.4)	0.89	1.05	(0.51–2.14)	0.62	1.33	(0.41–4.31)
Higher education	36 (31.5)	78 (68.5)		1			1	

*Presence of comorbidity*								
Yes	189 (50.5)	185 (49.5)	0.25	0.70	(0.39–1.28)	0.18	14.1	(0.28–696.18)
No	21 (42.0)	29 (58.0)		1			1	

*Stage in CKD*								
Stage 3	21 (39.6)	32 (60.4)		1				
Stage 4	15 (40.5)	22 (59.5)	0.93	1.03	(0.44–2.44)			
Stage 5	26 (50)	26 (50)	0.28	1.52	(0.70–3.301)			

*Length of service*								
≤1 year	35 (68.6)	16 (31.4)	0.001	2.87	(1.52–5.42)	0.21	1.82	(0.70–4.67)
2 years	45 (62.5)	27 (37.5)	0.01	2.19	(1.29–3.7)	0.73	1.14	(0.52–2.50)
≥3 years	130 (43.1)	171 (56.9)		1			1	

*Specialty*								
GP	20 (29.4)	48 (70.6)	<0.001	3.08	(1.72–5.51)	0.21	1.75	(0.72–4.26)
Internist	190 (53.3)	166 (46.7)		1			1	
Weight	61.4 (±9.2)	59.3 (±9.24)	0.02	1.02	(1.00–1.04)	0.22	0.97	(0.94–1.01)
Number of comorbidities	1.58 (±0.85)	2.84 (±1.0)	<0.001	3.99	(2.99–5.31)	0.01	1.65	(1.09–2.48) ^*∗*^
SrCr	3.02 (±2.30)	3.03 (±2.42)	0.96	0.99	(0.92–1.08)			
GFR	31.67 (±14.30)	33.74 (±15.02)	0.14	0.99	(0.97–1.00)	0.58	0.9	(0.97–1.01)
Number of medications	5.04 (±1.36)	3.02 (±1.18)	<0.001	3.29	(2.63–4.10)	<0.001	3.20	(2.28–4.49) ^*∗*^

^*∗*^ *p* < 0.05.

## Data Availability

All relevant data are in the manuscript. Additional data used to support the findings of this study are available from the corresponding author upon request.

## Supplementary Materials

Supplementary Materials Data collection tool .
