# Supplementary material for: Medicine Dose Adjustment Practice and Associated Factors among Renally Impaired Patients in Amhara Regional State, Ethiopia
Source: Int J Nephrol. 2021 Dec 1;2021:8238250. doi: 10.1155/2021/8238250 (PMC8654552; doi:10.1155/2021/8238250)
Supplement: Supplementary Materials — Data collection tool . [file 8238250.f1.docx]

# Annex: Data collection tool

## Section I. **Socio demographic characteristics of study participants**

| S.N | Variables | | Answer |
| --- | --- | --- | --- |
|  | Age (in years)  እድሜ (በአመት) | | _________________________ |
|  | Sex  ፆታ | | 1.Male 2.Female  ወንድ ሴት |
|  | Weight (in kg)  ክብደት (በኪሎግራም) | | _________________________ |
|  | Educational status  የትምህርት ደረጃ | | - Unable to read and write   ማንበብ እና መፃፍ የማይችል   - Literate with no formal education   መደበኛ ትምህርት ሳይኖረው መፃፍ እና ማንበብ የሚችል   - Primary education   የመጀመሪያ ደረጃ ትምህርት ያጠናቀቀ   - Secondary education   ሁለተኛ ደረጃ ትምህርት ያጠናቀቀ   - Higher education   ከፍተኛ ትምህርት ያጠናቀቀ |
|  | Residence  የመኖሪያ ቦታ | | 1. Rural 2.Urban  ገጠር ከተማ |
|  | Occupation  የስራ ሁኔታ | | - House wife   የቤት እመቤት   - Farmer   ግብርና   - Merchant   ንግድ   - Governmental employee   የመንግስት ሰራተኛ   - NGO employee   መንግስታዊ ያልሆነ ድርጅት ሰራተኛ   - Other   ሌላ |
| **Section II. Participants’ clinical characteristics and laboratory values** | | | |
|  | Diagnosis | |  |
|  | Stage in chronic kidney disease(CKD) patients | |  |
|  | Presence of comorbidity and complication | | 1.Yes 2.No |
|  | If yes, list of comorbidities | | _____________________________________ |
|  | Recent Serum creatinine (mg/dl) value | | ______________________________________ |
|  | Glomerular Filtration Rate/Creatinine clearance (ml/min) or eGFR: Cockcroft-Gault (CG) equation  Male: GFR ml/min= [(140-age (in year)) ×weight(kg)]  SrCr(mg/dl) ×72  Female: GFR ml/min= [(140-age (in year) ×weight(kg)]  SrCr(mg/dl) ×72  × 0.85(for females)  Reference ranges(eGFR≤60ml/min) | | __________________________________ |
| **Section III. Prescribers’ level of specialty and years of experiences** | | | |
|  | Specialty | | - General physician - Internist - Other |
|  | Length of service in years | |  |
| **Section IV. Medicines regimen prescribed- latest prescription** | | | |
| Ser. No | Name of medicines | Route of administration, dose and frequency, duration | |
|  |  |  | |
|  |  |  | |
|  |  |  | |
|  |  |  | |
|  |  |  | |
|  |  |  | |
|  |  |  | |
|  |  |  | |
|  |  |  | |
|  |  |  | |

**Section V: Evaluation of medicines dosage adjustment practice for those medicines guidelines recommended for dose adjustment.**

| Ser. No | Medicines Prescribed | Was the dose adjusted? (Yes/No) | If yes, was the dose adjusted correctly according to  recommendations  (Yes/No) | If not adjusted appropriately, was the inappropriateness arise from the dose or frequency? |
| --- | --- | --- | --- | --- |
|  |  |  |  |  |
|  |  |  |  |  |
|  |  |  |  |  |
|  |  |  |  |  |
|  |  |  |  |  |
|  |  |  |  |  |
|  |  |  |  |  |
|  |  |  |  |  |
|  |  |  |  |  |
|  |  |  |  |  |

## 
